# Structural Analysis of Redox-sensing Transcriptional Repressor Rex from *Thermotoga maritima*

**DOI:** 10.1038/s41598-018-31676-z

**Published:** 2018-09-05

**Authors:** Young Woo Park, Young Yoon Jang, Hyun Kyu Joo, Jae Young Lee

**Affiliations:** Department of Life Science, Dongguk University-Seoul, Ilsandong-gu, Goyang-si, Gyeonggi-do 10326 Republic of Korea

## Abstract

The cellular redox state of organisms continues to fluctuate during the metabolism. All organisms have various sensors that help detect and adapt to changes in the redox state. Nicotinamide adenine dinucleotides (NAD^+^/NADH), which are involved in various cellular metabolic activities as cofactors, have been revealed as the key molecules sensing the intra-cellular redox state. The Rex family members are well conserved transcriptional repressors that regulate the expression of respiratory genes by sensing the redox state according to the intra-cellular NAD^+^/NADH balance. Herein, we reported crystal structures of apo and NAD^+^- and NADH-bound forms of Rex from *Thermotoga maritima* to analyse the structural basis of transcriptional regulation depending on either NAD^+^ or NADH binding. The different orientation of the reduced nicotinamide group to helix α9 caused the rearrangement of N-terminal DNA binding domain, thus resulting in closed form of Rex to dissociate from cognate DNA. The structural data of Rex from *T. maritima* also support the previous redox-sensing mechanism models of Rex homologues.

## Introduction

Bacteria have adapted and responded to changing environmental oxygen levels for their survival by altering the transcriptional regulation of genes involved in respiratory pathways^1^. A reduction-oxidation (redox) reaction is a chemical reaction wherein the oxidation states of atoms are changed, involving a transfer of electrons between two molecules. Redox reactions are involved in many important biological processes, such as cellular respiration, photosynthesis, various biosyntheses, and enzymatic degradation. Intracellular redox state can be represented by the ratio of oxidized and reduced nicotinamide adenine dinucleotides (NAD^+^ and NADH, respectively). The NADs play an important role in cellular metabolism by acting as electron carriers^2^.

NADH is a major source of ATP through re-oxidizing to NAD^+^ by reduction of oxygen acting as an electron acceptor in electron transport chain^3^. The intracellular NAD^+^/NADH ratio changes when the NADH level is elevated because of low environmental oxygen levels or the inhibition of the electron transport chain^4^. Therefore, the NAD^+^/NADH ratio can be a critical indicator for the intracellular redox state. In other words, monitoring the continuous intracellular redox level using the NAD^+^/NADH ratio enables the sophisticated regulation of gene expressions involved in energy metabolism^5^.

The Rex family members are well-conserved transcription factors to control the respiratory pathway, which senses changes in the redox state according to the intracellular NAD^+^/NADH balance^6–8^. The Rex proteins negatively regulate the expression of the genes involved in catabolic metabolism, such as cytochrome *bd* oxidase (*cydABCD*), proton-translocating NADH dehydrogenase (*nuoA-N*), and NADH-linked fermentative lactate dehydrogenase (*lctP-ldh*), in the high level of NAD^+^^6,7^. The Rex homologues show an affinity for both oxidized NAD^+^ and reduced NADH but have a preference for the latter^6,9^. When the reduced NADH level is elevated, NADH is bound to a Rex protein, causing it to dissociate from the Rex DNA operator^8,9^.

A Rex protein comprises two domains; an N-terminal DNA binding domain containing a winged helix-turn-helix motif, and a C-terminal dimerization domain containing a NAD(H) binding site. A Rex protein exists as a homodimer and dimerization is significantly affected by the last helix of the C-terminal domain. The crystal structures of the Rex family have been determined from bacterial species, including *Thermus aquaticus*^8,10^, *Thermus thermophilus*^5^, *Bacillus subtilis*^9^, *Streptococcus agalactiae*, and *Thermoanaerobacter ethanolicus*^11^. Previous structural studies of the Rex family have described not only how conformation changes depending on whether they bind to NAD^+^ or NADH, but also how such conformational change induces the dissociation of cognate DNA from the Rex protein. Rex DNA operator sites of *Thermotoga maritima* have been identified and experimentally proved whether Rex from *T. maritima* (TmRex) could bind to cognate Rex binding sites depending on dinucleotide ligands using *in vitro* binding assays (*K*_*d*_ values of 5–27 nM)^12^. However, more investigation is needed on the detailed biochemical properties at the molecular level depending on NAD^+^ or NADH. To further understand the conformational changes of the Rex protein depending on the intracellular redox level, we determined crystal structures of apo and NAD^+^- and NADH-bound forms of the Rex proteins from *T. maritima* (TM0169). A structural comparison revealed that the transcriptional regulation of TmRex was controlled by NAD^+^/NADH cofactor inducing a conformational change of the dimeric structure.

## Results and Discussion

### Model building and quality

The initial structure of the apo TmRex was determined at 2.8 Å resolution using selenomethionine-based single-wavelength anomalous dispersion (SAD) data set collected at the selenium peak (0.9791 Å, Table S1). The refined model of SeMet-substituted TmRex gave R_work_ and R_free_ values of 23.86% and 26.75%, respectively. Subsequently, the native structure of apo TmRex was refined to 2.0 Å resolution with crystallographic R_work_ and R_free_ values of 20.40% and 25.83%, respectively. The refined model contained four TmRex subunits which formed two homodimers in an asymmetric unit. One dimer formed by subunits A and B was well ordered because of crystal contact stabilization, and the residues 4–208 in the subunit A and the residues 9–208 in the subunit B were interpretable. However, the N-terminal domains of the other dimer, formed by subunits C and D, were poorly ordered (average B-factors 79.6 Å^2^ and 73.1 Å^2^, respectively) (Fig. S1 and Table S2). Particularly, the glycine-rich loop comprising residues 55–63 was not visible due to the lack of electron-density maps. The NAD^+^-bound TmRex crystals were obtained by soaking NAD^+^ in apo crystals. The crystal structure of the NAD^+^-bound form was also determined at 2.2 Å resolution, and the NAD^+^ ligand was clearly observed in the 2Fo-Fc maps (Fig. S2). The structure of NAD^+^-bound TmRex was refined with crystallographic R_work_ and R_free_ values of 20.60% and 25.44%, respectively. One of the two dimers in the asymmetric unit is well defined, whereas the N-terminal domains of the other dimer was also disordered as shown in apo TmRex structure (Fig. S1 and Table S2). NADH-bound TmRex crystals were grown by co-crystallization. The structure of the NADH-bound form was determined at 2.45 Å resolution and refined to R_work_ and R_free_ of 21.53% and 24.73%, respectively. The refined model of NADH-bound TmRex includes 409 residues of the two independent Rex monomers and two NADH molecules in the asymmetric unit; the NADH molecules were clearly observed in the 2Fo-Fc maps (Fig. S2). All refined models for TmRex showed favoured or allowed regions of the Ramachandran plot. The detailed refinement statistics are summarized in Table 1.

**Table 1:** Data collection and refinement statistics.

Data set	Apo	NAD^+^-bound	NADH-bound
**Data collection statistics**			
Space group	P2_1_	P2_1_	P2_1_
**Unit-cell parameters**			
*a, b, c* (Å)	53.58, 88.46, 87.97	53.51, 89.45, 87.03	50.38, 76.84, 61.35
*α, β, γ* (°)	90.00, 96.81, 90.00	90.00, 96.76, 90.000	90.000, 90.60, 90.00
Wavelength (Å)	0.90000	0.97940	0.90000
Resolution (Å)	50.00–2.00 (2.03–2.00)	50.00–2.20 (2.24–2.20)	50.00-2.45 (2.49–2.45)
Number of observations	207850	154488	81389
Unique reflections	55075	41917	17292
Data completeness (%)	99.6 (99.3)	99.3 (99.6)	99.4 (100.0)
Redundancy	3.8 (3.8)	3.7 (3.8)	4.7 (4.8)
Averge I/σ(I)	18.2 (2.3)	16.6 (2.1)	20.0 (3.4)
R_merge_ (%)^a^	6.3 (55.9)	6.7 (44.8)	6.3 (41.2)
**Refinement statistics**			
Resolution (Å)	29.12-2.00	29.92-2.20	26.08-2.45
R_work_ / R_free_ (%)	20.40/25.83	20.60/25.44	21.53/24.73
NO. of non-H atoms	5867	6063	3133
Protein	5545	5821	3017
Ligands	24 (glycerol)	159 (NAD^+^)	100 (NADH)
Water	298	83	16
rmsd bonds (Å)	0.008	0.016	0.002
rmsd angles (°)	1.038	1.665	0.423
Average B-factor	48.56	64.44	72.72
Protein	48.52	64.24	72.76
Ligands	56.76	73.81	71.84
Water	49.25	60.45	71.45
**Ramachandran plot (%)**			
Favored	98.23	98.59	99.01
Allowed	1.77	1.41	0.99
Outliers	0	0	0

Values in parentheses refer to the highest resolution shell. ^a^*R*_merge_ = Σ_h_Σ_i_|*I*(*h*)_i_ − < *I*(*h*) > |/Σ_h_Σ_i_*I*(*h*)_i_, where *I*(*h*) is the _i_ntensity of reflection *h*, Σ_h_ is the sum over all reflections, and Σ_i_ is the sum over i measurements of reflection *h*.

### Overall structures of the *T. maritima* Rex

**Figure 1:**
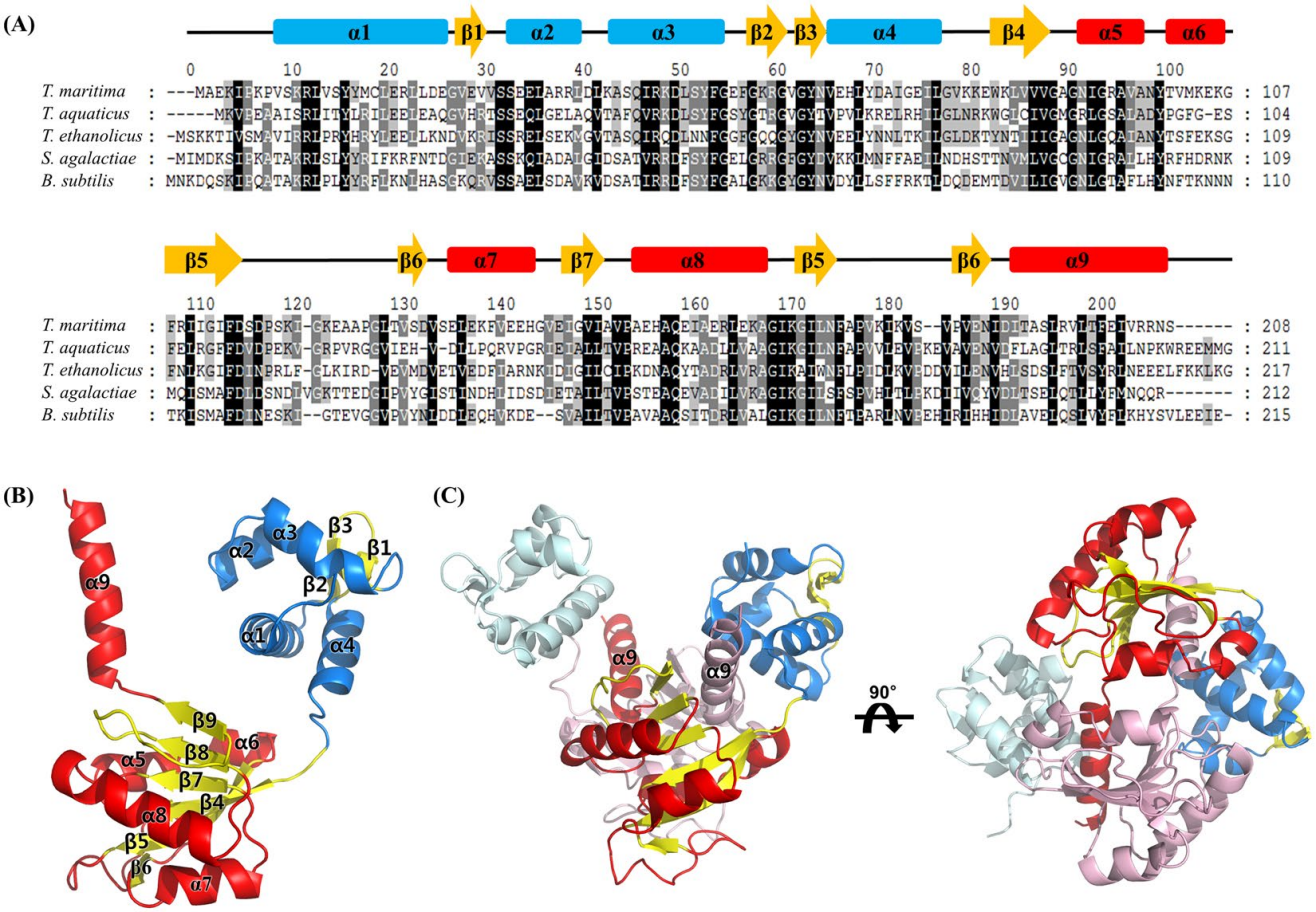
Multiple sequence alignment and overall structure of TmRex. (**A**) Multiple sequence alignment of TmRex with other Rex homologues. The secondary structures of TmRex are indicated above the sequence. The highly conserved and partially conserved residues are shaded in black and gray boxes, respectively. (**B**) The monomeric structure of apo TmRex. TmRex is composed of an N-terminal DNA binding domain and a C-terminal domain containing an extended helix α9. (**C**) The dimeric structure of apo form TmRex. The helix α9 is inserted into the inter-domain cleft between N- and C-terminal domains of the other subunit.

TmRex forms a homodimer, and each monomer was composed of two distinct domains: the N-terminal domain involved in DNA binding and the C-terminal domain involved in NAD binding, which were linked by a flexible loop (residues 78–83). The N-terminal domain (residues 1–77), which had a winged helix-turn-helix (HTH) motif, was folded into four α-helices and a hairpin loop. The C-terminal domain (residues 84–208), which contained the NAD(H) binding site, comprised six β-strands and five α-helices, showing a typical Rossman fold (Fig. 1B). The last helix α9 of the C-terminal domain was mainly responsible for dimerization through the formation of a hydrophobic interface by extending to between the N- and C-terminal domains of the other subunit (Fig. 1C). The residues of α9 (Ile192, Ala194, Leu196, Val198, Leu199, Phe201, Ile203, and Val204) contributed to the hydrophobic interface with the residues in the N-, and C-terminal domains, and the loop region (*Val14′, Tyr17′, Leu77′, Val102′, Phe108′, Trp83′, Leu85′, Try100′, Val150′, Leu174′, Phe176′, Ile190′, Pro191′)* of the other subunit (Fig. S3A). The short helix α5 in the C-terminal domain also contributed to dimerization by hydrogen bonding between the Asn92 residue and the main chain atoms of α5 helix in the other subunit (*Ala96′* and *Asn99′*) (Fig. S3B). The refined models of TmRex contained either two independent dimers (apo and NAD^+^-bound forms) or one dimer (NADH-bound form) in each asymmetric unit. Although the overall conformations of each domain in the asymmetric unit were similar (r.m.s.d. 0.5–1.6 Å), their position and orientation were varied due to the flexible linker loop (Fig. S4 and Table S3). There were little structural differences between apo and NAD^+^-bound dimeric forms (r.m.s.d. 0.7 Å), whereas NADH-bound form showed a large structural differences comparing with apo and NAD^+^-bound forms (r.m.s.d. 3.5–4.3 Å) (Fig. S5 and Table S4).

### NAD/NADH binding mode

The C-terminal domain of TmRex has the signature sequence of a P loop, which is common to dinucleotide binding protein, including Gly-X-Gly-X-X-Gly (Residues 89–94; Gly-Ala-Gly-Asn-Ile-Gly in TmRex). The dinucleotide ligands (NAD^+^ or NADH) are bound with 1:1 stoichiometry with each C-terminal domain near the dimer interface. The NAD(H) binding site of TmRex could be divided into two distinct parts: (1) the adenosine diphosphate (ADP) binding part, and (2) nicotinamide and N-ribose (connected with nicotinamide) binding part.

**Figure 2:**
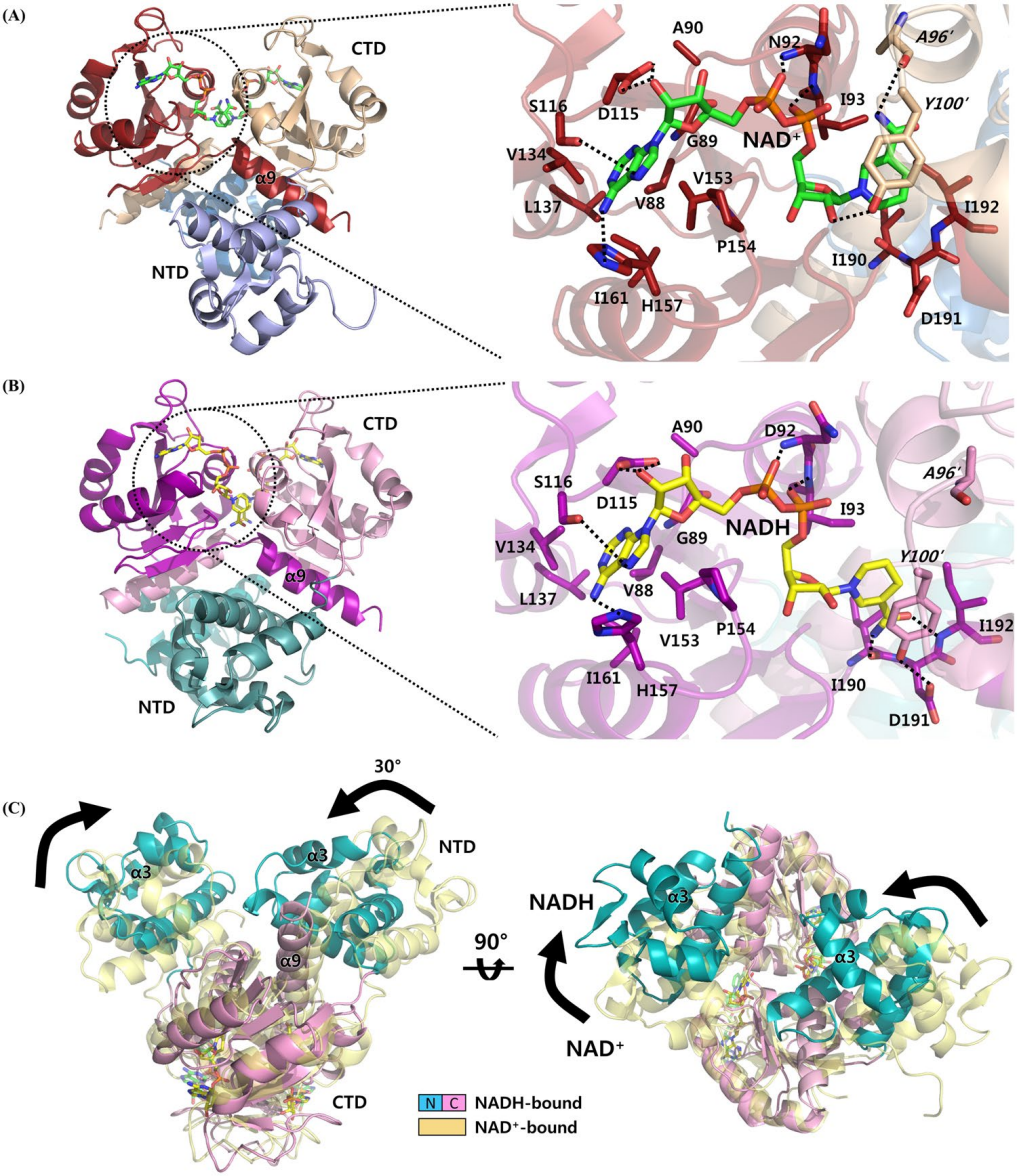
NAD^+^/NADH binding mode of TmRex. The oxidized and reduced dinucleotide ligands are bound to the C-terminal domain of TmRex. The hydrophobic residues mainly contribute to ligand binding and several hydrogen bonds also support their interaction, which is indicated by dotted lines. (**A**) NAD^+^-bound TmRex. Nicotinamide interacts with the main chain atom of *Ala96′* O, and N-ribose forms a hydrogen bond with the hydroxyl group of *Tyr100′*. (**B**) NADH-bound TmRex. The flipped nicotinamide interact with the main chain atoms of Ile190 and Ile192 at the N-terminus of the helix α9, and the hydroxyl group of *Tyr100′* forms a hydrogen bond with Asp191 Oγ. (**C**) Rearrangement of N-terminal domains depending on NAD^+^ or NADH binding. When NADH is bound to TmRex, the N-terminal domains tilt approximately 30°, allowing for higher proximity between DNA recognition helices (α3). The NAD^+^-bound form is shown in light yellow and the N- and C-terminal domains of the NADH-bound form are shown in pink and sky blue, respectively.

The adenosine moiety was buried in a hydrophobic pocket (Val88, Val134, Leu137, Val153, Pro154, and Ile161) and stabilized by hydrogen bonds with the residues Asp115, Ser116, and His157, as well as by van der Waals attractions involving a series of nonpolar residues (Val88, Gly89, and Ala90) of the P-loop (Fig. 2A,B). The pyrophosphate moiety of NAD(H) binds to the N-terminus of the helix α5 through hydrogen bonds with Asn92 N and Ile93 N atoms.

In both structures of TmRex bound to NAD^+^ and NADH, the ADP parts were well fixed at the ligand binding pocket and shared the same binding pattern, whereas nicotinamide and N-ribose moieties showed a different binding pattern. The nicotinamide ring of oxidized NAD^+^ is an aromatic organic compound composed of a conjugated planar ring system with a positively charged nitrogen. However, the charge of NADH is vanished by accepting two electrons, and the planarity of the nicotinamide ring is broken by forming only two double bonds. When the TmRex bound to NAD^+^, (1) the carboxamide group formed a hydrogen bond with the main chain atom O of *Ala96′*; (2) the nicotinamide ring stacked with the phenyl ring of a *Tyr100′* residue of a neighboring subunit and (3) the hydroxyl group of the *Tyr100′* residue formed a hydrogen bond with the 2*′* OH of the N-ribose moiety. In contrast to the NAD^+^-bound TmRex, the reduced nicotinamide of NADH was flipped by allowing the carboxamide group to form hydrogen bonds with the main chain atoms of Ile190 O and Ile192 N at the N-terminus of the helix α9. In addition, the hydroxyl group of the *Tyr100′* residue formed a hydrogen bond with the Asp191 residue in the helix α9. The differential binding of the nicotinamide ring between NAD^+^ and NADH caused the reorientation of the other subunit through the swapped C-terminal helix α9. Particularly, the N-terminal domains of each subunit tilted approximately 30° toward each other, resulting in higher proximity between the DNA recognition helices (α3) of the N-terminal domains from 35.7 Å to 29.5 Å (Fig. 2C). Because the rearranged N-terminal domains were too close to interact with the cognate DNA, the dramatic conformational change of the N-terminal domains by NADH binding allowed TmRex to dissociate from the DNA.

### Comparisons with other Rex family

We compared the structural and sequence similarities among the Rex proteins from various organisms using DALI server^13^ and Clustal Omega^14^. The five Rex homolouegs from *T. aquaticus*, *T. thermophilus*, *B. subtilis*, *S. agalactiae*, and *T. ethanolicus* were well matched with TmRex. Although each domain among the Rex homologues were structurally well aligned (r.m.s.d. 0.9–2.7 Å), their overall structures varied because of domain movement caused by the flexible loop (r.m.s.d. 1.9–4.0 Å) (Table S5).

**Figure 3:**
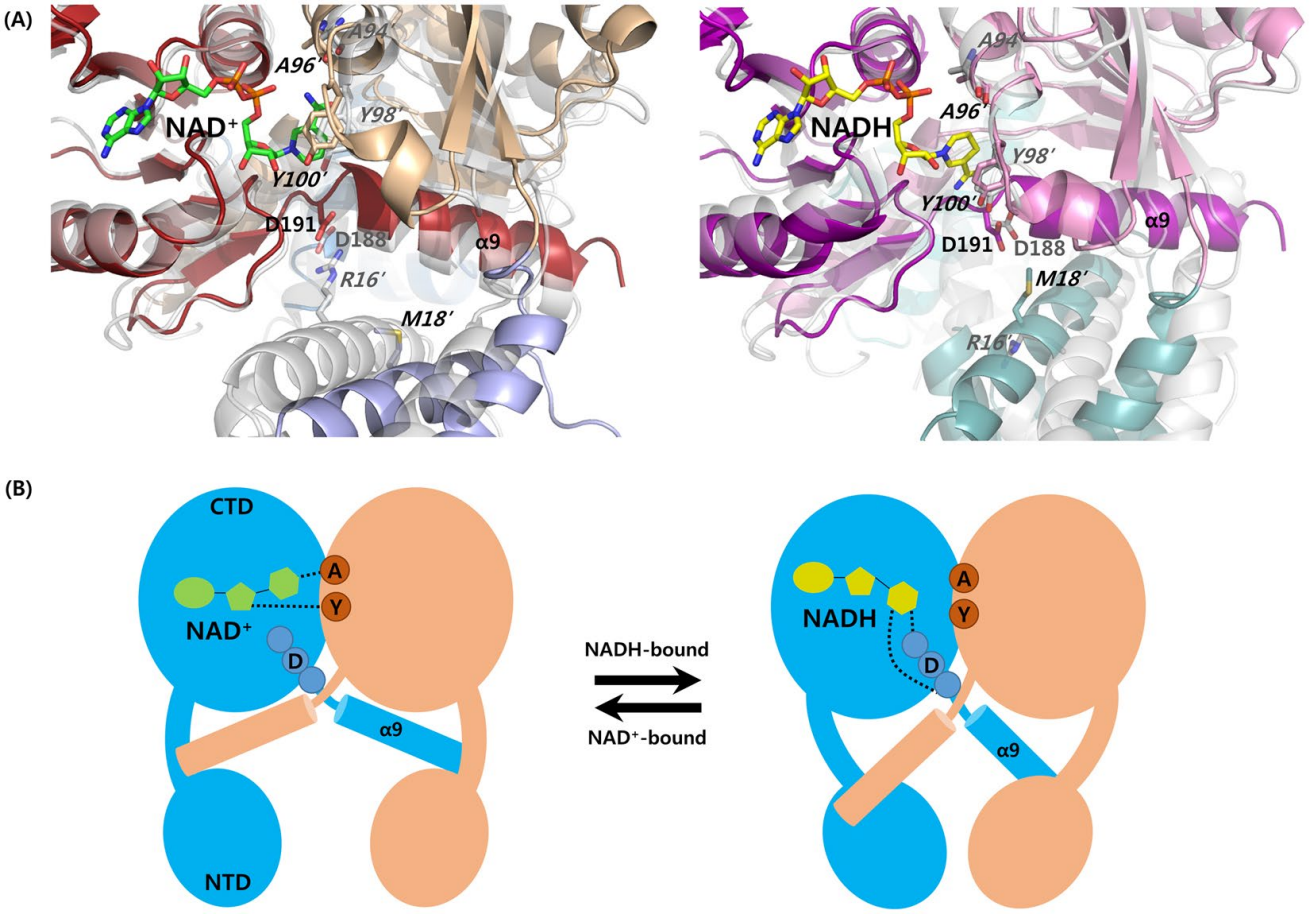
Comparisons with open and closed conformations between TmRex and *T. aquaticus* Rex and general mechanism model for NAD^+^/NADH sensing. (**A**) NAD^+^- and NADH- complex structures of TmRex are compared with those of the *T. aquaticus* Rex (gray). In NAD^+^-bound Rex structures (open conformation in left panel), the *T. aquaticus* Rex forms a salt bridge between the side chains of aspartate and arginine, whereas the aspartate residue of TmRex is not involved in any interaction because arginine of *T. aquaticus* is replaced by methionine. In NADH-bound Rex structures (closed conformation in right panel), NADH binding modes of the two Rex proteins are similar. (**B**) A general model for NAD^+^/NADH sensing by Rex homologous proteins. In open conformation (NAD^+^-bound), nicotinamide and N-ribose moieties interact with highly conserved residues *Ala′* and *Tyr′* of the other subunit, respectively. In closed conformation (NADH-bound), nicotinamide is flipped to interact with the main chain atoms at the N-terminus of the helix α9.

Previous researches of the *T. aquaticus* Rex have suggested that *Arg16′*, *Tyr98′*, and Asp188 were involved in the switching mechanism for open and closed forms of the Rex dimers depending on either NAD^+^ or NADH binding. Particularly, it was investigated that Asp188 played a key role in the sensing intracellular redox state. The Asp188 residue was salt-bridged to *Arg16′* in the open conformation when bound to NAD^+^, and the Asp188 residue interacted with the hydroxyl group of *Tyr98′* via a hydrogen bond in the closed conformation when bound to NADH. The residues were well conserved in the Rex homologues of *T. aquaticus*, *T. thermophilus*, *B. subtilis*, and *S. agalactiae*, whereas the arginine was replaced by *Met18′* in TmRex. Although the side chain of Asp192 of TmRex, which is equivalent to that of Asp188 in the *T. aquaticus* Rex, interacted with the hydroxyl group of *Tyr100′* in the closed conformation, the Asp192 was not involved in any interaction in the open conformation (Fig. 3A).

In the Rex structures of NAD^+^- or NADH-bound form, their adenosine moieties were buried at conserved hydrophobic pockets. In all the homologous structures, the oxidized nicotinamide interacted with equivalent residues of *Try100′* and the main chain atom of *Ala96′* in TmRex. The reduced nicotinamide formed hydrogen bonds with the main chain atoms of Ile190 and Ile192 in TmRex, which is consistently found in the Rex homologous structures. This common binding mode of nicotinamide in Rex repressors plays a key role in redox sensing regulation.

## Conclusion

This paper reported three crystal structures of TmRex: apo, NAD^+^-bound, and NADH-bound forms. TmRex composed of two distinct domains existed in the homodimeric form, and its overall structure was similar to those of other Rex homologues. In NAD(H)-bound TmRex, the dinucleotide ligands caused the differential orientation of the last helix α9 according to their redox state, which is commonly observed in other Rex homologues. When TmRex bound to NADH, the N-terminal domains were rearranged by the movement of the helix α9, resulting in a shorter distance between DNA recognition helices (α3) of the N-terminal domains. Several models of the redox-sensing mechanism have been proposed^8,11^. The structural data of TmRex also supported the previous models with a little modifications as shown in Fig. 3B. When Rex proteins bound to NAD^+^, the carboxamide group and N-ribose 2′ OH formed hydrogen bonds with the main chain atom O of alanine and the hydroxyl group of tyrosine, respectively. However, the carboxamide group of NADH formed hydrogen bonds with the main chain atoms at the N-terminus of the helix α9, and the hydrogen bond between N-ribose and tyrosine was broken. This structural model is widely applicable to other Rex homologous structures.

## Materials and Methods

### Expression and Purification of TmRex

The *rex* gene encoding TmRex was amplified from the genomic DNA of *Thermotoga maritima* by the polymerase chain reaction using oligonucleotide primers containing the artificial restriction endonuclease sites, NdeI and XhoI (forward primer: 5′-GGAATTC**CATATG**GCGGAAAAGATACCGAAG-3′ and reverse primer: 5′-CCG**CTCGAG**TCAAGAATTCCTCCTCACAATC-3′). The amplified DNA was cloned into expression vector, pET28b (+) (Novagen), and then, the recombinant plasmid was introduced into *E. coli* BL21 (DE3) star pLysS (Invitrogen) for protein overexpression. The integrity of the cloned gene was verified by DNA sequencing.

The transformed cells were grown up to OD_600_ ~0.5 at 310 K in Luria-Bertani medium containing 30 μg/ml chloramphenicol and kanamycin, respectively. The expression of the native TmRex was induced by the addition of 1 mM isopropyl-β-D-thiogalactopyranoside (IPTG) and the growth of the culture was continued at 303 K for 4 h. To get single-wavelength anomalous diffraction (SAD) data, selenomethionine (SeMet)-substituted TmRex was also expressed in the *E. coli* BL21 Star (DE3) pLysS cells in M9 salt medium supplemented with 2 μM MgSO_4_, 0.1 μM CaCl_2,_ 0.4% (*w/v*) glucose and a mixture of all amino acids at 40 mg/L except Asn, Ala, Gly, Cys, Pro and Met. When the OD_600_ of the cell culture reached ~ 0.5, 50 mg/L of Ile, Pro, Leu, and Val and 100 mg/L of Phe, Lys, and Thr were simultaneously added to inhibit Met synthesis^15^. After 15 min, 50 mg/L of Se-Met was added and the expression of SeMet-substituted TmRex was induced with 1 mM IPTG. The cells were continuously cultured at 303 K for 16 h. Each cell overexpressed native TmRex and Se-Met TmRex was collected by centrifugation at 2,300 g at 277 K for 15 min (Hanil Supra 22 K A500S-6 rotor) and immediately frozen at 193 K.

Because native and Se-Met TmRex were expressed with N-terminal hexa-histidine tag, a nickel affinity chromatography was applied. The cell pellets were re-suspended in the lysis buffer (500 mM NaCl, 20 mM Tris-HCl pH 8.0, 10% (*v/v*) glycerol, and 1 mM phenylmethylsulfonyl fluoride) and homogenized using an ultrasonic processor (Sonics ™ Vibra Cell VCX750) at 277 K. The cell lysate was centrifuged at 36,000 g for 1 h at 277 K and the insoluble fraction including cell debris was removed. The supernatant was loaded onto an immobilized-metal-affinity-chromatography (IMAC) column charged nickel ion (GE Healthcare). The native TmRex was eluted with the elution buffer (500 mM NaCl, 20 mM Tris-HCl pH 8.0, 10%(*v/v*) glycerol, and 300 mM imidazole). The TmRex was further purified by size exclusion chromatography using Superdex 200 gel-filtration column (GE Healthcare) pre-equilibrated with gel-filtration buffer (200 mM NaCl, 2 mM MgCl_2_, 10 mM Tris-HCl at pH 8.0, 5%(*v/v*) glycerol and 1 mM dithiothreitol (DTT)). The purity of TmRex was assessed by polyacrylamide gel electrophoresis in the presence of 0.1%(*w/v*) sodium dodecyl sulfate. The histidine tag of the SeMet-substituted TmRex was removed by PreScission protease after identical process of Ni^+^-affinity chromatography performed in purification step of the native TmRex, and then finally purified by size-exclusion chromatography.

### Crystallization and X-ray data collection

The purified native and SeMet-substituted proteins of TmRex were concentrated up to 36 mg/mL for crystallization using a Centrifugal Filter (Millipore). The crystallization was carried out using a sitting-drop vapour diffusion method at 296 K using 96-well CrystalQuick plates (Greiner Bio-one) and commercial screens from Hampton Research, Qiagen and Emerald BioSystems. Each sitting-drop was prepared by mixing equal volume of the concentrated protein and reservoir solution (0.75 μl, respectively). The crystals of the apo TmRex were initially obtained in several reservoir solution containing polyethylene glycol (PEG) 3,350, which were further optimized to 200 mM potassium chloride and 20%(*w/v*) PEG 3,350. The NADH complex crystals were obtained by co-crystallization with 1 mM NADH and were grown at 200 mM ammonium chloride and 20%(*w/v*) PEG 3,350. The redox state of NAD^+^/NADH was monitored at a wavelength of 340 nm using a spectrophotometer (NanoDrop, ThermoFisher Scientific). The crystals of SeMet-substituted TmRex were obtained in the condition containing PEG 8,000, and were further optimized to 100 mM HEPES pH 7.0 and 8%(*w/v*) PEG 8,000.

Prior to data collection, each crystal of apo, NAD(H) complex and SeMet-substituted TmRex was transferred into a cryo-protectant solution containing the reservoir solution with 25%(*v/v*) glycerol, and was then flash-frozen in a liquid nitrogen stream. X-ray diffraction data of apo and NADH complex crystals of TmRex were collected at 100 K with an RAYONIX MX300HE CCD detector using synchrotron radiation on BL44XU beamline of the SPring-8 in Japan. The crystals were exposed to X-ray for 1.0 s per image, and 280 frames (apo) and 230 frames (NADH complex) were obtained with each 1.0° oscillation. X-ray diffraction data of the NAD^+^ complex and SeMet-substituted crystals of TmRex were collected at 100 K with a Pilatus 6 M detector and ADSC Q315r CCD detector, respectively, using synchrotron radiation on beamline 5 C at the Pohang Accelerator Laboratory (PAL) in Republic of Korea. In order to collect diffraction data of the NAD^+^-bound crystal, the apo crystals were soaked with 1 m*M* NAD^+^ for 10 h. The NAD^+^ complex and SeMet-substituted crystals were exposed to X-ray for 1.0 s and 1.5 s per image, and 200 frames (NAD^+^ complex) and 100 frames (SeMet-substituted) were respectively recorded. All data were processed and scaled using *DENZO* and *SCALEPACK* from the *HKL-2000* software^16,17^.

### Structure determination and refinement

In order to determine the initial structure of the apo TmRex, the SAD data from SeMet-substituted TmRex crystal were collected. Eight of twelve possible selenium sites were found and the SAD-phased electron-density map was interpreted using *AutoSol* and *AutoBuild* from *PHENIX* program suite^18^. Subsequently, the native structure of apo TmRex was solved by molecular replacement (*PHASER MR*, *CCP4 program* suite)^19^, and was refined to 2.0 Å resolution. The structures of NAD^+^ and NADH complexes were solved by molecular replacement using the apo structure and were refined to 2.2 Å and 2.45 Å resolution, respectively. All TmRex structures were manually built and refined by *Coot*^20^ and *PHENIX*^21^ program suite. The refined models were finally evaluated by MolProbity^22^.

#### Accession numbers

Coordinate and structure factors have been deposited in the Protein Data Bank (PDB): apo TmRex, PDB ID, 5ZZ5; NAD^+^-bound TmRex, PDB ID, 5ZZ6; NADH-bound TmRex, PDB ID, 5ZZ7.

## Electronic supplementary material

Supplementary Information

## Data Availability

All data are fully available without restriction.
