# Supplementary material for: Structural Analysis of Redox-sensing Transcriptional Repressor Rex from Thermotoga maritima
Source: Sci Rep. 2018 Sep 5;8:13244. doi: 10.1038/s41598-018-31676-z (PMC6125430; doi:10.1038/s41598-018-31676-z)
Supplement: Supplementary file 1 — Supplementary Information [file 41598_2018_31676_MOESM1_ESM.pdf]

Structural Analysis of Redox-sensing Transcriptional Repressor Rex from *Thermotoga maritima*

Young Woo Park[1], Young Yoon Jang[1], Hyun Kyu Joo[1], Jae Young Lee[1]*

[1] Department of Life Science, Dongguk University-Seoul, Ilsandong-gu, Goyang-si, Gyeonggi-do, 10326, Republic of Korea

*Corresponding author

E-mail: jylee001@dongguk.edu

Supplementary Table 1. SAD data collection statistics

Data set	Se-Peak (Apo)
**Data collection**	
Wavelength (Å)	0.9791
Space group	$P4_1$
Unit-cell parameters	
a, b, c (Å)	100.58, 100.58, 127.34
α, β, γ (°)	90.00, 90.00, 90.00
Resolution (Å)	50.00-2.80 (2.85-2.80)
Number of observations	130327
Unique reflections	31052
Completeness (%)	99.3 (99.6)
Redundancy	4.2 (4.2)
Averge I/σ(I)	34.59 (2.64)
R_{merge} (%)[a]	6.1 (70.4)
**SAD phasing**	
Se sites	11 (12 possible sites)
Figure of merit	0.79 (0.87 after density modification)

Values in parentheses refer to the highest resolution shell. [a] $R_{merge} = \Sigma_h \Sigma_i |I(h)_i - \langle I(h) \rangle| / \Sigma_h \Sigma_i I(h)_i$, where $I(h)$ is the intensity of reflection h, Σ_h is the sum over all reflections, and Σ_i is the sum over i measurements of reflection h.

Supplementary Table 2. Average B-factors of TmRex structures.

Apo	Subunit A			Subunit B			Subunit C			Subunit D		
	full	NTD	CTD	full	NTD	CTD	full	NTD	CTD	full	NTD	CTD
Average B-factor (Å^2)	46.8	50.4	44.7	43.9	48.7	40.7	53.6	79.6	47.9	54.2	73.1	46.5

NAD^+-bound	Subunit A			Subunit B			Subunit C			Subunit D		
	full	NTD	CTD	full	NTD	CTD	full	NTD	CTD	full	NTD	CTD
Average B-factor (Å^2)	67.0	68.8	65.8	57.6	59.8	56.1	68.1	77.2	62.4	64.7	85.9	55.0

NADH-bound	Subunit A			Subunit B		
	full	NTD	CTD	full	NTD	CTD
Average B-factor (Å^2)	76.8	73.7	79.0	68.6	65.1	70.9

Supplementary Table 3. Structural comparisons among subunits in each TmRex.

apo	Subunit B			Subunit C			Subunit D		
	Full	NTD	CTD	Full	NTD	CTD	Full	NTD	CTD
Subunit A (R.m.s.d., Å)	3.6	1.1	0.7	1.5	-	0.6	2.4	1.2	0.5
NAD^+-bound	Subunit B			Subunit C			Subunit D		
	Full	NTD	CTD	Full	NTD	CTD	Full	NTD	CTD
Subunit A (R.m.s.d., Å)	3.9	1.0	0.5	2.2	1.6	0.6	2.4	1.1	0.6
NADH-bound	Subunit B								
	Full			NTD			CTD		
Subunit A (R.m.s.d., Å)	3.1			1.0			0.5		

Supplementary Table 4. Structural comparisons among TmRex dimeric structures

		Apo		NAD^+- bound	
		Dimer 1	Dimer 2	Dimer 1	Dimer 2
Apo	Dimer 1	-			
	Dimer 2	2.5	-		
NAD^+- bound	Dimer 1	0.7	2.5	-	
	Dimer 2	3.1	0.7	2.9	-
NADH- bound	Dimer 1	3.6	3.9	3.5	4.3

Supplementary Table 5. Structural comparisons of TmRex and other homologues.

apo	TmRex (R.m.s.d., Å)											
	Subunit A			Subunit B			Subunit C			Subunit D		
	Full	NTD	CTD	Full	NTD	CTD	Full	NTD	CTD	Full	NTD	CTD
T. aquaticus Rex (3IKV)	2.3	2.0	1.6	3.4	1.9	1.4	1.9	-	1.6	1.9	0.9	1.3

NADH-bound	TmRex (R.m.s.d., Å)					
	Subunit A			Subunit B		
	Full	NTD	CTD	Full	NTD	CTD
T. aquaticus Rex (1XCB)	3.4	2.3	1.6	2.4	1.6	1.8

NADH-bound	TmRex (R.m.s.d., Å)					
	Subunit A			Subunit B		
	Full	NTD	CTD	Full	NTD	CTD
T. thermophilus Rex (2DT5)	3.5	1.8	1.6	2.2	1.8	1.7

apo	TmRex (R.m.s.d., Å)											
	Subunit A			Subunit B			Subunit C			Subunit D		
	Full	NTD	CTD	Full	NTD	CTD	Full	NTD	CTD	Full	NTD	CTD
T. ethanolicus RSP (3WG9)	2.3	1.8	1.5	3.3	2.7	1.5	2.1	-	1.5	2.2	1.1	1.4

NAD^+-bound	TmRex (R.m.s.d., Å)											
	Subunit A			Subunit B			Subunit C			Subunit D		
	Full	NTD	CTD	Full	NTD	CTD	Full	NTD	CTD	Full	NTD	CTD
T. ethanolicus RSP (3WGG)	2.0	1.8	1.6	3.6	2.4	1.4	2.7	1.6	1.6	2.4	1.6	1.4

NADH-bound Rex	TmRex (R.m.s.d., Å)					
	Subunit A			Subunit B		
	Full	NTD	CTD	Full	NTD	CTD
T. ethanolicus RSP (3WGH)	3.7	1.9	1.3	2.2	1.8	1.4

NAD^+-bound	TmRex (R.m.s.d., Å)											
	Subunit A			Subunit B			Subunit C			Subunit D		
	Full	NTD	CTD	Full	NTD	CTD	Full	NTD	CTD	Full	NTD	CTD
S. agalactiae Rex (3KEO)	2.1	1.3	2.0	4.0	1.9	1.9	3.3	2.2	2.2	2.7	1.3	2.0

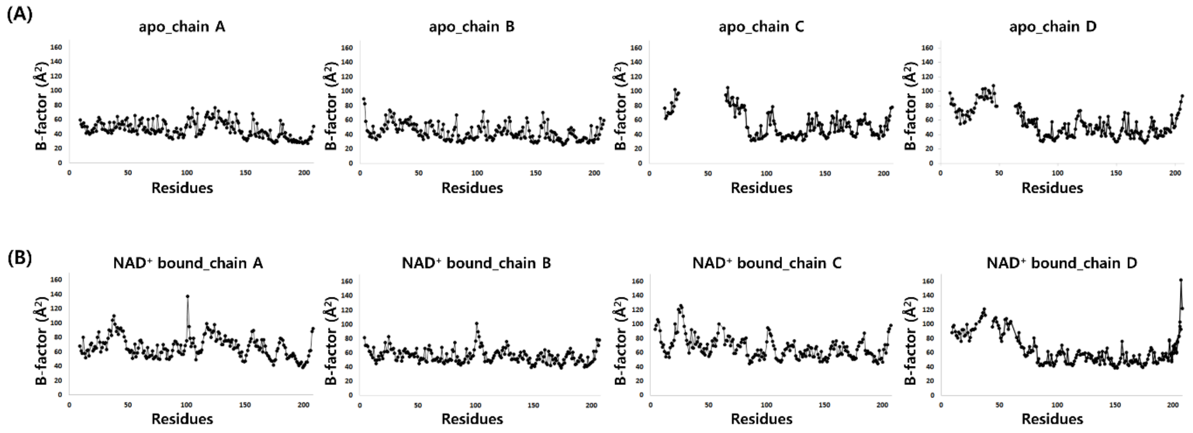

Supplementary Figure 1. B-factor plots of apo and NAD$^+$-bound TmRex. Four subunits of apo (A) and NAD$^+$-bound (B) TmRex in an asymmetric unit were drawn according to the B-factor of Cα atoms, respectively. In both apo and NAD$^+$-bound TmRex, one dimer formed by subunits A and B was well ordered, whereas the N-terminal domains of the other dimer, formed by subunits C and D, were poorly ordered by high B-factor.

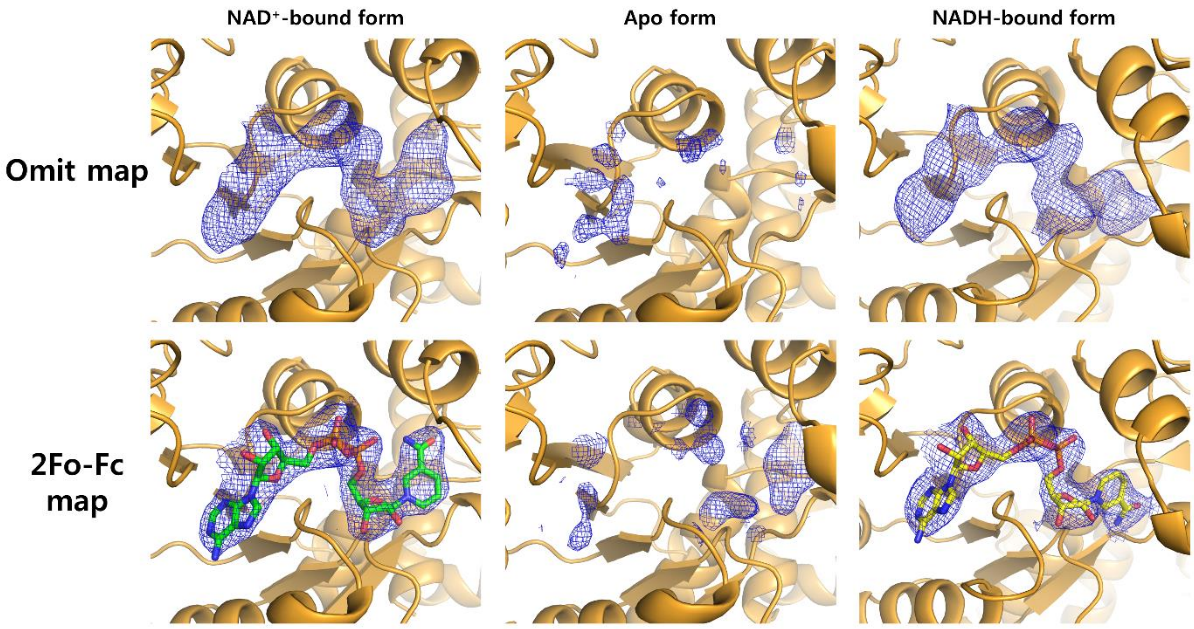

Supplementary Figure 2. Electron density maps around Dinucleotide ligand binding sites of TmRex. Omit maps (upper) and 2Fo-Fc maps (bottom) were illustrated around dinucleotide ligand binding sites. Each map was generated at contoured level 2.0 σ for omit maps and 1.0 σ for 2Fo-Fc maps within 2.0 Å.

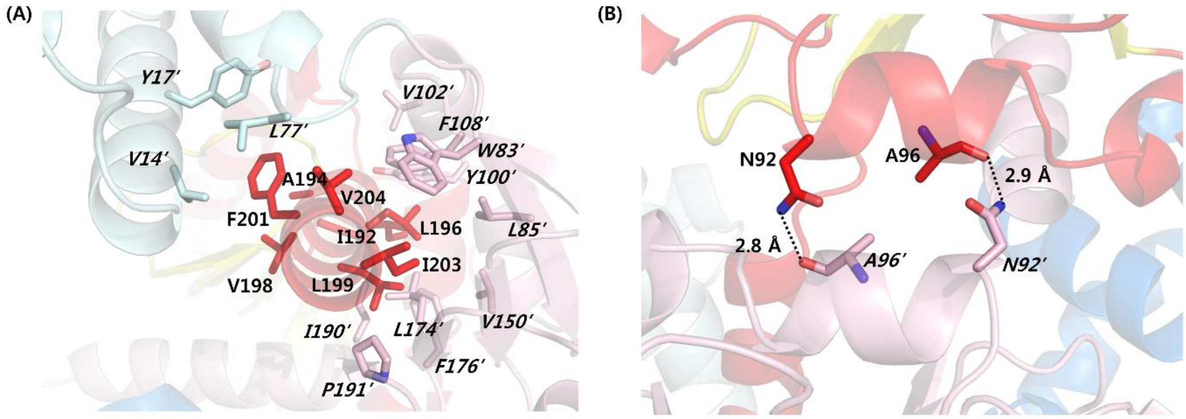

Supplementary Figure 3. Dimerization regions of TmRex. (A) The last helix α9 of the C-terminal domain is mainly responsible for dimerization by formation of a hydrophobic interface with the N- and C-terminal domains of the other subunit. (B) The short helix α5 in the C-terminal domain also contributes to dimerization by hydrogen bonding between the Asn92 residue and the main chain atoms of α5 helix in the other subunit (Ala96′ and Asn99′).

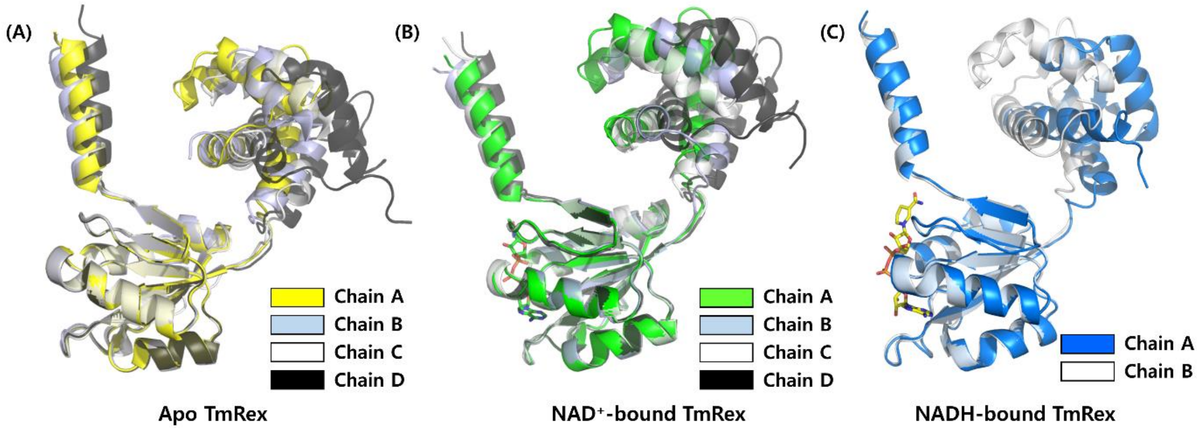

Supplementary Figure 4. Structural alignments of subunits in each TmRex structure. Although overall conformations of each domain in the asymmetric unit of apo and NAD^+- and NADH-bound TmRex are similar, their position and orientation are varied due to flexible loop connecting the N-terminal domain and the C-terminal domain.

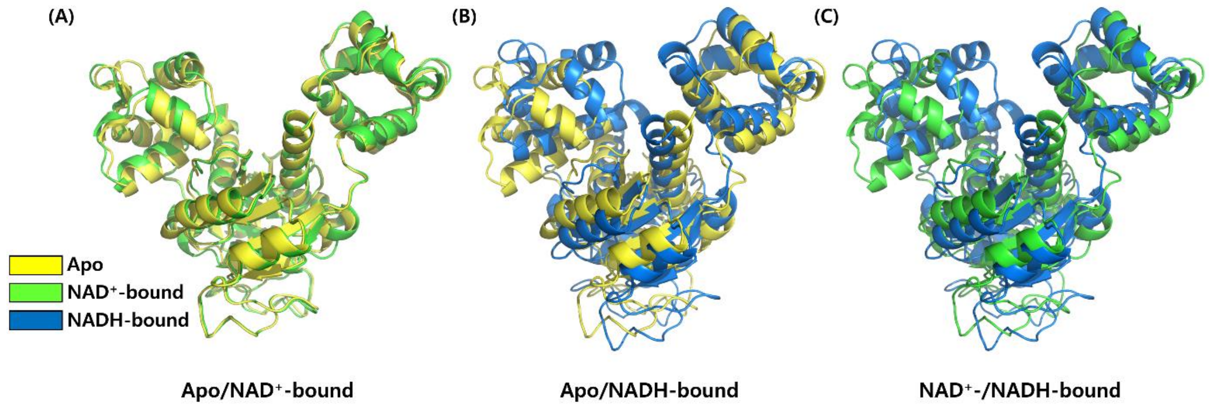

Supplementary Figure 5. Structural alignments between dimeric TmRex structures. The TmRex dimeric structures were compared with each other. (A) There were little structural differences between apo and NAD^+-bound forms, whereas NADH-bound form showed a large structural differences comparing with apo and NAD^+-bound forms (B) and (C).
